# Infestation of humans and non-human primates with *Cordylobia rodhaini* (Diptera: Calliphoridae) in a ‘hotspot’ of furuncular myiasis

**DOI:** 10.1017/S0031182025100875

**Published:** 2025-10

**Authors:** Tony L. Goldberg

**Affiliations:** Department of Pathobiological Sciences, School of Veterinary Medicine, University of Wisconsin-Madison, Madison, WI, USA

**Keywords:** blowfly, DNA barcoding, ecology, Lund’s fly, morphology, non-human primates, zoonosis

## Abstract

Lund’s fly, *Cordylobia rodhaini* (Calliphoridae), is an African blowfly considered to be an uncommon cause of furuncular myiasis. Far less is known about *C. rodhaini* than about the more frequently reported tumbu fly, *Cordylobia anthropophaga*. From 2011 to 2020, fly larvae were collected and analysed from 11 independent infestations of wild non-human primates and 10 independent infestations of humans (including 1 from this author) in Kibale National Park, Uganda. All 21 larvae were identified morphologically and genetically as *C. rodhaini*. Larvae from non-human primates were on average 4·5 times larger than larvae from humans. Non-human primates had empty furuncles, indicating recent egress of mature third instar larvae and completion of the larval stage of the lifecycle; however, eastern chimpanzees (*Pan troglodytes schweinfurthii*) were photographed removing larvae from furuncles of grooming partners. A total of 4 closely related mitochondrial haplotypes were identified, 2 of which were shared by larvae from humans and non-human primates. Genetic variation within *C. rodhaini* from this single location was comparable to that within other calliphorid species. Non-human primates may play a larger role in the maintenance of *C. rodhaini* than previously known, and in certain forested locations *C. rodhaini* may be the predominant cause of furuncular myiasis. The sylvatic lifecycle of *C. rodhaini* may explain its differentiation from *Cordylobia anthropophaga*, which has a peridomestic lifecycle. In general, these findings shed new light on how myiasis-causing flies can adapt to different ecological settings and be regionally rare but locally abundant.

## Graphical abstract

**Figure:**
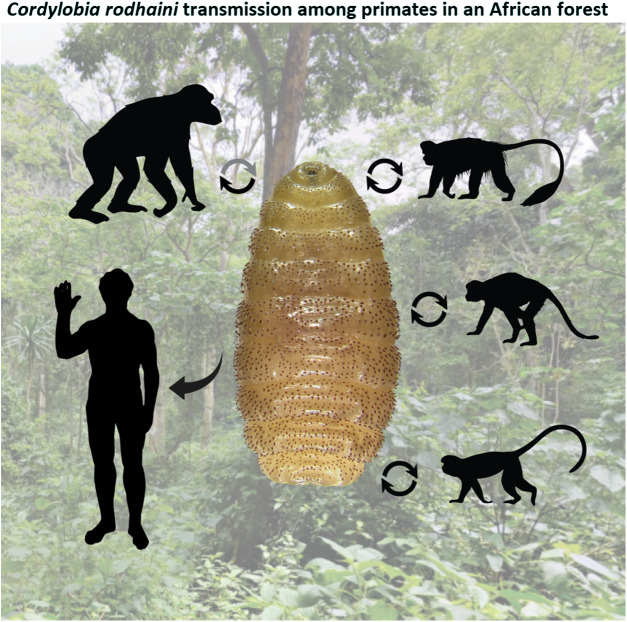

## Introduction

Furuncular myiasis is caused by taxonomically and geographically diverse dipteran flies whose larvae infest the skin of vertebrate hosts (Baird et al. 1989; Solomon et al. 2016; Mathison et al. 2022). As larvae develop, they create painful boils (‘furuncles’) that can ooze serosanguineous fluid, which also contains larval faeces and can become secondarily infected with bacteria (Günther, 1971; Geary et al. 1999). Furuncular myiasis is usually mild and self-limiting (if unpleasant), but it can be severe in some cases (Pampiglione et al. 1992; Wade et al. 2019; Muñoz et al. 2021; Jesuyajolu and Jesuyajolu, 2022). In the tropics, the most commonly reported taxa are the human botfly *Dermatobia hominis* Linnaeus Jr. 1781 (Oestridae) in South and Central America (Villalobos et al. 2016; Ragi et al. 2021) and blowflies of the genus *Cordylobia* Grünberg 1903 (Calliphoridae) in Africa (Jallow et al. 2024).

Furuncular myiasis in Africa has been described in the medical literature since the 1800s (Zumpt, 1965). In the early 1900s, scientists in endemic countries made detailed morphological descriptions of the flies, surveyed domestic animals and wildlife to identify reservoirs, and conducted experimental infestations (Zumpt, 1965). Since then, most publications have been case reports involving travellers returning from Sub-Saharan Africa (Jallow et al. 2024). Such patients typically present with symptoms initially misdiagnosed as insect bites, pyoderma, inflamed epidermoid cysts or similar conditions, and patients often receive antibiotics before the actual cause is identified (Grassi et al. 2016; Solomon et al. 2016; Muñoz et al. 2021; Jallow et al. 2024). The number of cases of furuncular myiasis exported from Africa is almost certainly higher than the number reported in the literature (Jallow et al. 2024). Case reports may be biased towards ‘dramatic’ infestations involving, for example, extraordinary intensities (in some cases over 100 larvae) or disquieting anatomic locations (Brodin and Rodhain, 1910; Pampiglione et al. 1992; Grassi et al. 2016; Wade et al. 2019; Jesuyajolu and Jesuyajolu, 2022). Studies of the ecology of these flies have been all but absent since initial investigations in the early 1900s, which shaped prevailing paradigms about their natural history (Zumpt, 1965).

Here, I describe a study of fly larvae causing furuncular myiasis in Kibale National Park, Uganda (‘Kibale’, hereafter). Kibale is a tropical forest in the biodiverse Albertine Rift of East Africa, and its diversity and density of non-human primates (‘primates’ hereafter) is exceptional (Oates et al. 1990; Plumptre et al. 2007). Kibale is also a ‘hotspot’ for furuncular myiasis, as evidenced by the high risk of contracting the condition for visitors to the park (personal observation) and by its representation in case reports involving international travellers returning with the condition (Jallow et al. 2024). Among the community of research personnel at Kibale and among residents of communities adjacent to the park, ‘ebimonde’ (singular ‘ekimonde’) or ‘mango flies’ (the local colloquial terms for the causative larvae in Rutooro and English, respectively) are a well-known and common nuisance. However, to my knowledge, no formal studies of these flies have previously been performed in Kibale.

Using field observations and fly larvae collected over a decade from people and primates in Kibale, this study sheds new light on the ecology of a lesser-known cause of furuncular myiasis, Lund’s fly (*Cordylobia rodhaini* Gedœlst 1909). Female flies of this species and other members of the genus *Cordylobia* lay eggs on soil contaminated with urine or faeces, on fruit, or on wet surfaces. The eggs hatch, and first instar larvae undergo questing behaviour in response to movement or warmth (Günther, 1971). After attaching to a host, larvae burrow into the skin, where they develop to the third instar stage over approximately 1 to 2 weeks (Rodhain and Bequaert, 1916; Geary et al. 1999). Mature larvae excyst, fall to the ground, burrow approximately 4–8 cm into the soil, pupate and emerge as adults approximately 25 days later (Rodhain and Bequaert, 1916; Günther, 1971; Geary et al. 1999). Relevant to human infestation, females have a vexing predilection for ovipositing on wet clothing hung to dry (Zumpt, 1965; Pampiglione et al. 1992; Hall and Smith, 1993). Data presented herein suggest that aspects of this lifecycle may have evolved in response to specialization on primates. In general, host preference may be a decisive factor in the evolutionary divergence of species within the genus *Cordylobia*, providing an informative example of how myiasis-causing flies can adapt to different ecological settings and be regionally rare but locally abundant.

## Materials and methods

### Field studies

Larvae were collected from primates that had been anesthetized and released back to their social groups as part of separate studies of health and conservation (Goldberg et al. 2012; Paige et al. 2015); details of animal capture and sampling have been described previously (Lauck et al. 2011). When a furuncle was found during physical examination, steady digital pressure was applied to its base until the larva was expelled. Larvae were immediately placed in RNAlater nucleic acid stabilization solution (Thermo Fisher, Waltham, MA, USA) to facilitate future studies of both DNA and RNA and were stored at −20°C. Only 1 larva per primate infestation was used for molecular analysis, to avoid pseudoreplication (*i.e.* analysing multiple larvae from the same oviposition event).

Larvae from humans (*Homo sapiens* Linnaeus 1758) were obtained through unsolicited donations from individuals who had heard of this author’s peculiar interest in these parasites. When larvae were received, they were immediately placed in RNAlater and stored as above. As with primates, only 1 larva per human infestation was used for molecular analysis.

### Laboratory studies

Larvae were photographed using methods previously described for other parasitic fly larvae (Friant et al. 2022). Briefly, a JVC KY-F75U digital camera attached to a Leica Z16 APO dissecting microscope with apochromatic zoom objective and motor focus drive was used to capture images of larvae from each host species sampled. Larvae were illuminated by a LED ring light and dual gooseneck fibre optic illuminators. A Syncroscopy Auto-Montage system (Synoptics, Cambridge, UK) and associated software was then used to create image stacks from white-balance-corrected individual images. Photoshop v. 25.4.0 (Adobe, San Jose, CA, USA) was used to crop stacked (montaged) images and to normalize exposure and colour balance. Larvae were identified morphologically using a dichotomous key (Zumpt, 1965).

DNA was extracted from larvae using the DNeasy Blood & Tissue Kit (Qiagen, Hilden, Germany) according to the manufacturer’s protocol. DNA barcoding was performed by amplifying and sequencing a 657 base pair region of the cytochrome oxidase subunit 1 (*cox1*) gene, as previously described (Ramírez-Martínez et al. 2021). Resulting *cox1* sequences were aligned with MUSCLE (Edgar, 2004) to homologous calliphorid *cox1* sequences in GenBank as of October 6 2025. Where more than 1 published sequence was available for a species, the 2 maximally divergent sequences (measured as percent nucleotide difference) were included to capture the range of known intraspecific variation. A phylogenetic tree was inferred using the maximum likelihood method implemented in PhyML 3.3 (Guindon et al. 2010) with smart model selection (Lefort et al. 2017) and 1000 bootstrap replicates to assess statistical support for clades.

## Results

### Field studies

**Figure 1.**
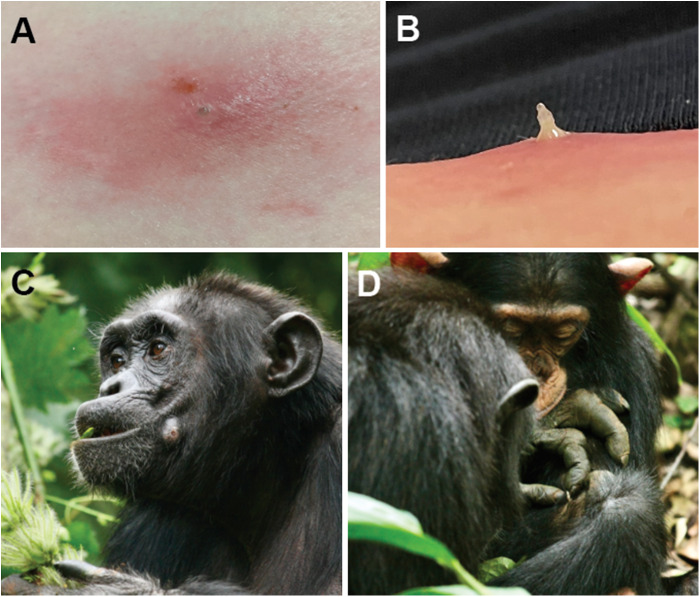
Myiasis-causing fly larvae in humans and chimpanzees, Kibale National Park, Uganda. (A) Furuncle on the arm of a human; note oozing fluid. (B) Larva in the process of being expressed from human furuncle using manual pressure. (C) Chimpanzee with large furuncle on left cheek. (D) Chimpanzee removing larva from furuncle on the arm of grooming partner. Photo credits: Jessica Rothman (A and B); Nicholas Brazeau/Kibale Chimpanzee Project (C and D).

From 2010 to 2014, larvae were collected from 11 primates of out of 114 primates sampled, for a period prevalence of 9·6% (95% confidence interval 5·3%–16·6%). The 11 infested primates included 9 red colobus monkeys (*Piliocolobus tephrosceles* Elliot 1907), 1 red-tailed guenon (*Cercopithecus ascanius* Audebert 1799) and 1 black-and-white colobus monkey (*Colobus guereza* Rüppell 1835). These 11 primates had between 1 and 9 larvae, with an average of 1·9 larvae per infestation (standard error of the mean = 1·7), although this is likely an underestimate due to the difficulty of finding lesions on parts of the body covered with hair. All larvae collected were removed from separate furuncles and appeared morphologically identical. Several primates had empty/healing furuncles, indicating prior infestations and natural emergence of mature third instar larvae. Primates not sampled but observed as part of other research projects also showed visual evidence of furuncular myiasis on hairless regions of the body (face, hands, feet and anogenital region), including olive baboons (*Papio anubis* Lesson 1827), grey-cheeked mangabeys (*Lophocebus albigena* J. E. Gray 1850) and eastern chimpanzees (*P. t. schweinfurthii* Giglioli 1872). Remarkably, eastern chimpanzees were photographed removing larvae from each other’s furuncles during mutual grooming sessions (Figure 1C and D).

From 2011 to 2020, 10 larvae were collected and analysed from humans (including 1 from the left axillary region of this author). Donors were all adults and were an approximately equal mix of local and international research personnel. Donors did not generally specify the anatomic locations of their infestations, although accounts suggested that arms, armpits, torso, legs, face, neck, head and genitals are all possible. Within those anatomic locations, infestations tended to occur in places where clothing comes into firm contact with skin (e.g. the elastic straps of undergarments). All larvae were expressed manually from furuncles when they were still relatively small (Figure 1A and B). Unlike primates, humans had no evidence of past infestations that had matured, nor did anyone report successful attempts to allow larvae to mature (although some tried, in the pursuit of knowledge).

### Laboratory studies

**Figure 2.**
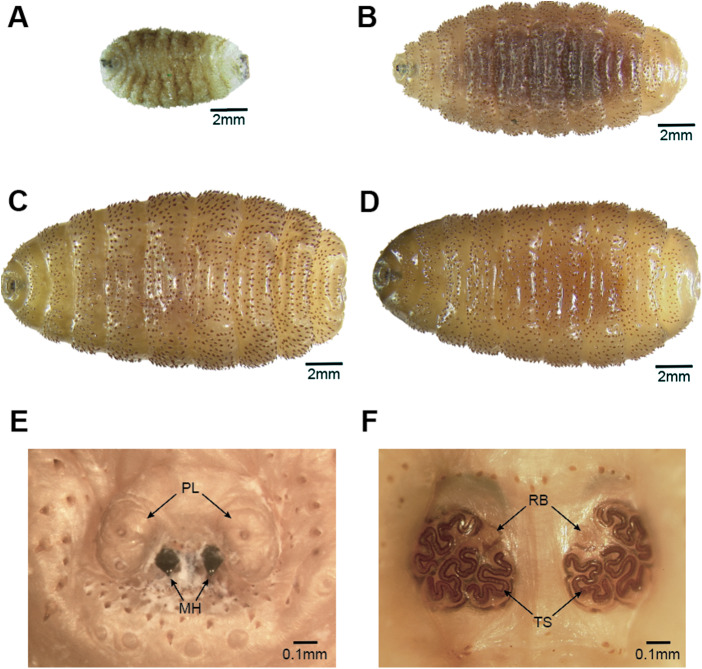
Montaged images of *Cordylobia rodhaini* third instar larvae from Kibale National Park, Uganda. (A) Larva *in toto* from this author (a human), ventral aspect. (B) Larva *in toto* from a black-and-white colobus monkey, ventral aspect. (C) Larva *in toto* from a red colobus monkey, ventral aspect. (D) Larva *in toto* from a red-tailed guenon, ventral aspect. (E) Anterior aspect showing pseudo cephalic lobes (PL) and mouth hooks (MH) and (F) posterior aspect showing tortuous slits of the posterior peritremes (TS) and respiratory buttons (RB), of larva from a red colobus monkey.

Larvae had morphological characteristics consistent with third instar members of the genus *Cordylobia*, including large, backwardly directed curved spines densely arranged along the exterior (Figure 2A–D) and 2 prominent pseudo cephalic lobes (Figure 2E; Hall and Smith, 1993; Zumpt, 1965). All larvae were identified as *C. rodhaini* based on the presence of tortuous slits of the posterior peritremes (Figure 2F), which is a morphological feature that distinguishes *C. rodhaini* from *Cordylobia ruandae* Fain 1953 and the tumbu fly, *C. anthropophaga* Blanchard & Bérenger-Feraud 1872 (Gedœlst, 1910; Zumpt, 1965). Larvae from primates were 4·5 times larger than larvae from humans (16·2 ± 2·3 mm vs. 3·6 ± 1·1 mm, respectively), consistent with size variation documented in third instar larvae of this genus (Zumpt, 1965), and this difference was statistically significant (Student’s t = 15·69, 19 degrees of freedom; *P* < 0·001).

**Figure 3.**
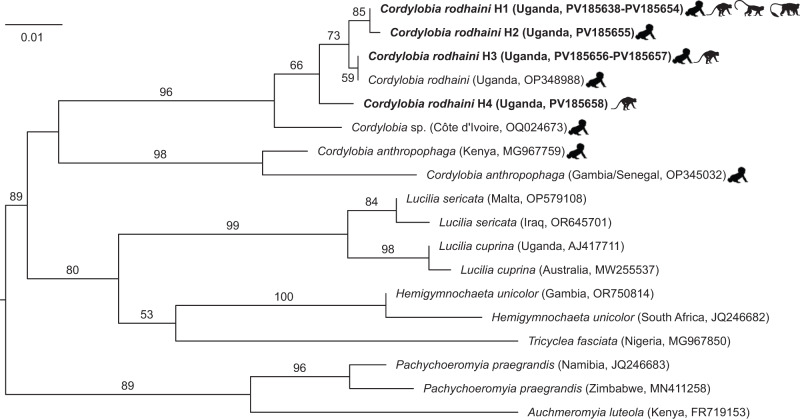
Maximum likelihood phylogenetic tree of flies in the family Calliphoridae. The tree is based on a 574-position trimmed nucleotide sequence alignment of the mitochondrial cytochrome oxidase subunit 1 gene containing 111 variable positions and a GTR + I model of molecular evolution. Sequences were chosen to represent the maximum degree of variation within each species. Taxon names are followed (in parentheses) by country of origin and GenBank accession number. For the clade representing the genus *Cordylobia*, silhouettes indicate the host(s) from which specimens were obtained. The 4 haplotypes identified in this study (H1-H4) are highlighted in bold. The tree is midpoint rooted. Numbers beside branches indicate bootstrap values (percent) based on 1000 replicates; only values ≥ 50% are shown. The scale bar indicates nucleotide substitutions per site.

Sequencing of *cox1* was successful for all larvae. Sequences represented 4 haplotypes (sequences differing by at least 1 nucleotide). Haplotype 1 (GenBank PV185638–PV185654) was present in larvae from 8 humans, 7 red colobus monkeys, 1 red-tailed guenon and 1 black-and-white colobus monkey. Haplotype 2 (GenBank PV185655) was present in a larva from a human. Haplotype 3 (GenBank PV185656–PV185657) was present in larvae from 1 human and 1 red colobus monkey. Haplotype 4 (GenBank PV185658) was present in a larva from a red colobus monkey. A phylogenetic tree (Figure 3) shows these haplotypes to form a clade with a sequence of *C. rodhaini* from a morphologically confirmed third instar larva recovered from a traveller returning to Poland from Uganda in 2018 (Biernat et al. 2025). Haplotype 3 is, in fact, identical to this sequence. Haplotypes 1, 2 and 4 differ from the Polish sequence by 0·523%, 0·697% and 1·22%, respectively. All haplotypes differ from an unidentified *Cordylobia* sp. from Côte d’Ivoire (GenBank OQ024673) by 1·92% to 2·61%. The clade containing the 4 haplotypes from this study and the unidentified *Cordylobia* sp. from Côte d’Ivoire is sister to the clade representing *C. anthropophaga.* Sequences representing the genus *Cordylobia* cluster separately from those of other myiasis-causing calliphorids (Figure 3). The maximum nucleotide sequence difference within *C. rodhaini* collected in Kibale (1·52%) falls within the range of maximum nucleotide sequence differences calculated for other species in the analysis (from 0·35% for *Lucilia cuprina* Wiedemann 1830 to 2·96% for *C. anthropophaga*; Figure 3).

## Discussion

Gedœlst first described *C. rodhaini* in 1905 based on a third instar larva collected in August 1902 from the arm of a ‘commandant Lund’ (Gedœlst, 1905). Gedœlst made detailed morphological descriptions but was unable to assign the larva to species, instead coining the provisional designation ‘larve de Lund’ (Gedœlst, 1905); thus, ‘Lund’s fly’. Gedœlst further described the species in 1909 from specimens of first, second and third instar larvae and pupae provided by Rodhain and Bequaert in Léopoldville, Belgian Congo (now Kinshasa, Democratic Republic of the Congo; Gedœlst, 1910; Rodhain and Bequaert, 1916). Surcouf (1914) and Rodhain and Bequaert (1916) provided morphological descriptions of female and male adult specimens, respectively, placing the species in a separate genus, *Stasisia*, which was subsequently synonymized with the genus *Cordylobia* (Zumpt, 1965). Brodin and Rodhain (1910) first described furuncular myiasis caused by *C. rodhaini*.

Rodhain and Bequaert conducted the only prior investigation of the lifecycle and natural history of *C. rodhaini* (Rodhain and Bequaert, 1916; Zumpt, 1965), to this author’s knowledge. They documented that adults are crepuscular, remaining mostly immobile from 8 am to 5 pm (Rodhain, 1915). They successfully established a breeding colony and conducted experimental infestations of guinea pigs (*Cavia porcellus* Linnaeus 1758), which proved to be suboptimal hosts (Rodhain and Bequaert, 1916). Similarly, they achieved only limited success with self-infestation experiments (Rodhain and Bequaert, 1916). However, they observed normal maturation of 5 of 7 larvae in 12–15 days in a naturally infested African pouched rat (*Cricetomys gambianus* Waterhouse 1840; Rodhain and Bequaert, 1916). They also reported local knowledge of high infestation rates in *C. gambianus* and various duikers (forest antelopes of the genus *Cephalophus* Smith, 1827; Rodhain and Bequaert, 1916). These observations account for the still-pervasive view that pouched rats and duikers are important reservoir hosts. Later, Zumpt (1965) listed various shrews, duikers, squirrels, rats, a gerbil and the mona monkey (*Cercopithecus mona* Schreber 1774) as hosts of second and/or third instar larvae, but the source of this information is unclear. Although this list of hosts has appeared repeatedly in the subsequent literature, the identity of the true natural reservoir(s) of *C. rodhaini* should be considered an open question.

Currently, *C. rodhaini*, is considered an uncommon cause of furuncular myiasis in people, given that it accounts for only 10% of reports of human African cutaneous myiases in the literature, versus 66% of reports attributable to *C. anthropophaga* (Jallow et al. 2024). Therefore, it was surprising that all 21 larvae analysed from people and primates in Kibale were identified as *C. rodhaini*. This result was also surprising because the local English term for the parasite, ‘mango fly’, refers to *C. anthropophaga*, also known as the ‘tumbu fly’ (Blacklock and Thompson, 1923). Inquisitive visitors to Kibale have long assumed *C. anthropophaga* to be the cause of their unpleasant experiences, based primarily on published reports and internet searching, which reveal this species to be the most common regionally. The origin of the term ‘mango fly’ is unclear, but it may refer to the attraction of *C. anthropophaga* to fruit (Zumpt, 1965), such as would be found underneath mango trees (*Mangifera indica* Linnaeus 1753). Despite the rarity of mango trees within the forests of Kibale, *C. rodhaini* is abundant there. Rodhain and Bequaert (1916) successfully attracted *C. rodhaini* adults using various fruit juices, making it likely that *C. rodhaini* forages on the variety of wild fruits that Kibale wildlife (including primates, duiker and African pouched rats) consume, as well as excretions of these animals beneath fruiting trees.

Of case reports of *C. rodhaini* infestations in the literature as of October 6 2025, 3 of 13 involved visitors to Uganda, with other reports spanning Sub-Saharan Africa, from Ethiopia to Cameroon (Jallow et al. 2024). Of these 3 reports, 2 identify Kibale as the location where the infestation was acquired (Pezzi et al. 2015; Wade et al. 2019), and the 3rd report does not specify a location within Uganda (Veraldi et al. 2014). Uganda has 10 national parks, 5 of which feature tropical forests (Plumptre et al. 2019; Gessa et al. 2024), but individuals who frequent Uganda’s other forested parks rarely report furuncular myiasis. A confluence of host availability, spatial overlap between people and primates and favourable environmental conditions may account for why Kibale appears to be a ‘hotspot’ for *C. rodhaini* and furuncular myiasis. Of note, humans are dead-end hosts for *C. rodhaini* and *C. anthropophaga*, in that people do not generally tolerate larval maturation (Zumpt, 1965 and personal observation, although anecdotal reports suggest that a few past visitors to Kibale have succeeded in hosting larvae until spontaneous emergence). Chimpanzees, our closest living relatives, apparently share this intolerance (as they do many other traits), as evidenced by their behaviour of removing larvae from each other’s furuncles (Figure 1D). In addition to ironing one’s clothes thoroughly, measures to control human infestation might include alternatives to open pit latrines and open rubbish pits. The predilection of *C. rodhaini* for faeces, urine and fruit might attract adult flies to these peridomestic features, thus increasing human infestation risk.

Primates appear to play a more important role in the lifecycle of *C. rodhaini* than previously suspected. Kibale is known for its diversity and density of primates (Oates et al. 1990; Plumptre et al. 2007). Larvae were recovered from 9·6% of primates sampled, and furuncular lesions were observed in other primates. Primates also had empty furuncles, indicating successful maturation of third instar larvae (Rodhain and Bequaert, 1916). Human and primate activity spaces overlap in Kibale, creating ample opportunities for transmission (Paige et al. 2017). Pouched rats and duiker do occur in Kibale (Kingdon et al. 2013a, b), so these species may also contribute to the local abundance of *C. rodhaini*, but Kibale is not known for a high density and biomass of these species, as it is for primates (Oates et al. 1990; Plumptre et al. 2007). Specialization of myiasis-causing flies on primates is not without precedent, as evidenced by *Cuterebra baeri* (Shannon and Greene, 1926), which preferentially infests primates in the Neotropics and occasionally humans (Shannon and Greene, 1926; Rondón et al. 2023).

*C. rodhaini* differs from *C. anthropophaga* in ways that suggest ecological niche differentiation. *C. anthropophaga* typically infests domestic dogs (*Canis familiaris* Linnaeus 1758) and other domestic species, having a lifecycle adapted to human peridomestic spaces (Günther, 1971; Jallow et al. 2024). By contrast, *C. rodhaini* infests wild forest animals but is not known to infest dogs or other domestic animals, having a sylvatic lifecycle (Zumpt, 1965). As mentioned above, both species have a predilection for ovipositing on wet clothing hung to dry (Zumpt, 1965; Pampiglione et al. 1992; Hall and Smith, 1993). This behaviour would seem an inefficient way to infest giant rats, duiker or other hosts implicated as *C. rodhaini* reservoirs. However, it might reflect a misdirected strategy to deposit eggs on the (often wet) vertical trunks and leaves of trees, such as occur in tropical forests like Kibale, and with which primates come into extensive contact.

A total of 4 haplotypes were found in larvae from humans and primates in Kibale, 1 of which was identical to a previously published sequence from a morphologically confirmed *C. rodhaini* larva (Biernat et al. 2025). Of these four haplotypes, 2 (H1 and H3) were shared between primates and humans, and the other 2 were closely related. These data suggest that some human infestations likely originate from larvae maturing in primates, in which case furuncular myiasis caused by *C. rodhaini* can be considered a primate zoonosis. The 4 haplotypes identified in this study were sampled from a single geographically defined population. Nevertheless, these haplotypes were as genetically variable as those from other calliphorid species. Additional characterization of *C. rodhaini* across its entire range would probably reveal even greater genetic variation. These results support the suspicion of Biernat et al. (2025) that intraspecific genetic variation in *C. rodhaini* exists, but they do not resolve whether the divergent sequence from Côte d’Ivoire lies within or outside the degree of variation demarcating the species (Figure 3). The Côte d’Ivoire sequence could represent a geographically and genetically divergent population of *C. rodhaini*, or it could represent another related taxon.

Studies of the ecology of myiasis-causing flies are difficult because they require a sustained field presence, facilities for rearing and experimentation and extensive sampling of putative hosts in biodiverse ecosystems. In this light, it is important to note that the current study was opportunistic, and sampling was not systematic. Nevertheless, the results point to a significant role of primates in the maintenance of *C. rodhaini.* More generally, this study sheds light on how parasitic flies can adapt to specific ecological conditions and be regionally rare but locally abundant. It remains unclear what health and fitness effects, if any, *C. rodhaini* has on wild primates. A study of *C. baeri* infestation in mantled howler monkeys (*Alouatta palliata* Gray 1849) in Panama showed that increasing parasite burden was associated with decreased serum albumin levels and increased mortality, with the overall effect of limiting population growth (Milton, 1996). Given the painful nature of the clinical condition, the risk of secondary bacterial infection, and the intensities of infestation that can sometimes occur, it is reasonable to posit that the negative effects of *C. rodhaini* on African primates may be consequential.

## Data Availability

All *cox1* sequences were deposited in the NIH National Center for Biotechnology Information GenBank database, accession numbers PV185638–PV185658.
